# Rate of Progression of Aortic Stenosis in Patients With Cancer

**DOI:** 10.3389/fcvm.2021.644264

**Published:** 2021-03-18

**Authors:** Katia Bravo-Jaimes, Nicolas L. Palaskas, Jose Banchs, Nadia I. Abelhad, Alveena Altaf, Sushanth Gouni, Juhee Song, Saamir A. Hassan, Cezar Iliescu, Anita Deswal, Syed Wamique Yusuf

**Affiliations:** ^1^Division of Cardiology, Department of Medicine, University of Texas Health Science Center at Houston, Houston, TX, United States; ^2^Division of Internal Medicine, Department of Cardiology, University of Texas MD Anderson Cancer Center, Houston, TX, United States; ^3^Department of Medicine, University of Texas Health Science Center at Houston, Houston, TX, United States; ^4^Division of Biostatistics, University of Texas MD Anderson Cancer Center, Houston, TX, United States

**Keywords:** aortic stenosis, cancer, echocardiography, progression, cyclophosphamide

## Abstract

Patients with cancer and aortic stenosis (AS) are exposed to several factors that could accelerate the progression of AS. This study aimed to determine the cumulative incidence of AS progression and associated factors in these patients. This retrospective cohort study included patients with cancer, mild or moderate AS and at least two echocardiograms 6 months apart between 1996 and 2016 at MD Anderson Cancer Center. AS progression was defined by an increase in mean gradient of 20 mmHg or peak velocity of 2 m/s by spectral Doppler echocardiography or as requiring aortic valve replacement. Univariate and multivariable Fine-Gray models to account for the competing risk of death were used. One hundred and two patients were included and median follow-up was 7.3 years. Overall, 30 patients (29%) developed AS progression, while 48 (47%) died without it. Yearly rate of mean gradient change was 4.9 ± 3.9 mmHg and yearly rate of peak velocity change was 0.23 ± 0.29 m/s for patients who developed AS progression. In the univariate analysis, coronary artery disease (CAD), dyspnea, prevalent cyclophosphamide and beta-blocker use were associated with AS progression. In multivariable analysis, CAD and prevalent cyclophosphamide use for the time interval of more than 3 years of follow-up remained significantly associated with increased cumulative incidence of AS progression. In conclusion, patients with mild or moderate AS and cancer are more likely to die before having AS progression. AS progression is associated with CAD and prevalent cyclophosphamide use.

## Introduction

Cancer and cardiovascular disease are the two leading causes of mortality in the United States (1). Patients with both cancer and aortic stenosis (AS), are exposed to factors that could potentially accelerate AS progression, including chest radiation (2, 3) and cardiotoxic drugs such as anthracyclines (4). These have been noted to produce *de-novo* AS via valve leaflet thickening, fibrosis, retraction and calcification (4, 5). However, the impact that they may have on AS progression has not been studied. Current guidelines for the general population recommend surveillance echocardiography every 3 to 5 years in patients with mild AS and every 1 to 2 years in those with moderate AS, in the absence of symptoms of left ventricular (LV) dysfunction (6). These recommendations may not apply to the specific cancer population since the rate of AS progression in cancer patients is unknown. Therefore, this study aimed to determine the cumulative incidence and factors associated with the cumulative incidence of AS progression in a contemporary cohort of cancer patients.

## Materials and Methods

### Study Population

This retrospective cohort study included all adults with mild or moderate AS who underwent treatment at MD Anderson Cancer Center between January 1, 1996 to December 31, 2016 and had at least two transthoracic echocardiograms (TTEs) 6 months apart. The severity of AS was defined per current societal echocardiographic guidelines (7). Patients with prior aortic valve replacement (AVR), severe AS, or LV ejection fraction <50% at baseline were excluded. The University of Texas MD Anderson Institutional Review Board approved the protocol and informed consent was waived due to the retrospective nature of the study. The study protocol conforms to the ethical guidelines of the 1975 Declaration of Helsinki as reflected in a priori approval by the institution's human research committee.

### Clinical Data

Baseline clinical data was collected from the time where mild or moderate AS was first detected by echocardiography. Clinical parameters which included baseline demographics, comorbidities, symptoms, cancer type, cancer stage, and medication usage were collected through manual chart review of electronic medical records. Advanced cancer was defined as stage greater than T2 and/or N1 and/or M1 as well as any malignancy considered refractory, relapsing, or recurrent and cancer treated with transplantation (8). Variables related to chemotherapy [use of anthracyclines, cyclophosphamide, taxanes, tyrosine kinase inhibitors, vascular endothelial growth factor (VEGF) inhibitors or human epidermal growth factor receptor 2 (HER2) antagonists] and chest radiation were obtained and classified with respect of the date of baseline TTE as prevalent (before baseline TTE) or incident (after baseline TTE). i.e., prevalent cyclophosphamide was defined as cyclophosphamide administration prior to baseline TTE.

### Echocardiographic Imaging

All patients underwent comprehensive TTEs with commercially available instruments (Philips Medical Systems, NA, Bothell, Washington; General Electric Medical Systems, Milwaukee, Wisconsin; and Siemens Medical Solutions USA, Inc., Malvern, Pennsylvania) as part of their standard diagnostic evaluation (i.e., pre-chemotherapy or when clinically indicated). LV ejection fraction was calculated using Simpson's biplane method by the European Association of Cardiovascular Imaging and the American Society of Echocardiography recommendations (9). LV outflow tract diameter was measured in a standard fashion on zoomed parasternal long-axis views, and the LV outflow tract peak velocity and velocity-time integral were calculated using Pulse-Wave doppler, according to societal guidelines (7, 9, 10). Additionally, peak velocity, velocity-time integral and mean resting aortic valve gradients were recorded to quantify AS severity as per societal guidelines (7, 9, 10). A pedhoff probe was used to measure the highest possible velocities.

### Outcomes Measurement

AS progression was defined by an increase in the aortic valve mean gradient of 20 mmHg or peak velocity of 2 m/s by spectral Doppler echocardiography confirmed by two cardiologists (S.W.Y., N.P.) or as requiring AVR (which was assessed by the heart valve team at the referral hospital). Survival status until the last day of follow-up was assessed through patient's individual electronic medical records.

### Statistical Analysis

Patient characteristics were summarized by descriptive statistics. Univariate and multivariable Fine-Gray models to account for the competing risk of death were used to identify factors associated with increased cumulative incidence of AS progression (11). Time to progression was defined as the time interval from the initial TTE to the TTE where AS progression was first detected or AVR was performed. In those who did not progress, time to death or last follow-up was used. The event of interest was progression, and death without progression was considered as a competing risk event. Three progression statuses were determined as progressed, died, or alive without progression. Cumulative incidence function (CIF) accounting for a competing risk was estimated by a non-parametric method and Gray's test was used to compare CIFs by different subgroups. The proportional hazards assumption was checked by testing statistical significance of interaction terms involving failure time [i.e., the interaction between a function of time (the log of time) and covariates] (12). In the presence of violation of proportionality, the time-dependent coefficients were added in the Fine-Gray model. Age group, prevalent cyclophosphamide, and statin use were included in the multivariable model. For those who developed AS progression and received AVR, the median survival time was calculated as the time when 50% of the subgroup died according to the reverse Kaplan-Meier method (13). Two sensitivity analyses using rate of progression as a continuous variable were performed with the goal of identifying if certain subgroups (divided by age, sex, cancer type, stage, chest radiation) had faster progression. The first analysis used mean gradient change divided by the time from baseline TTE to last follow-up TTE and the second used jet velocity change divided by the time from baseline TTE to last follow-up TTE. A *p* < 0.05 was used to indicate statistical significance. SAS 9.4 (SAS Institute INC, Cary, NC) was used for statistical analysis.

## Results

**Figure 1:**
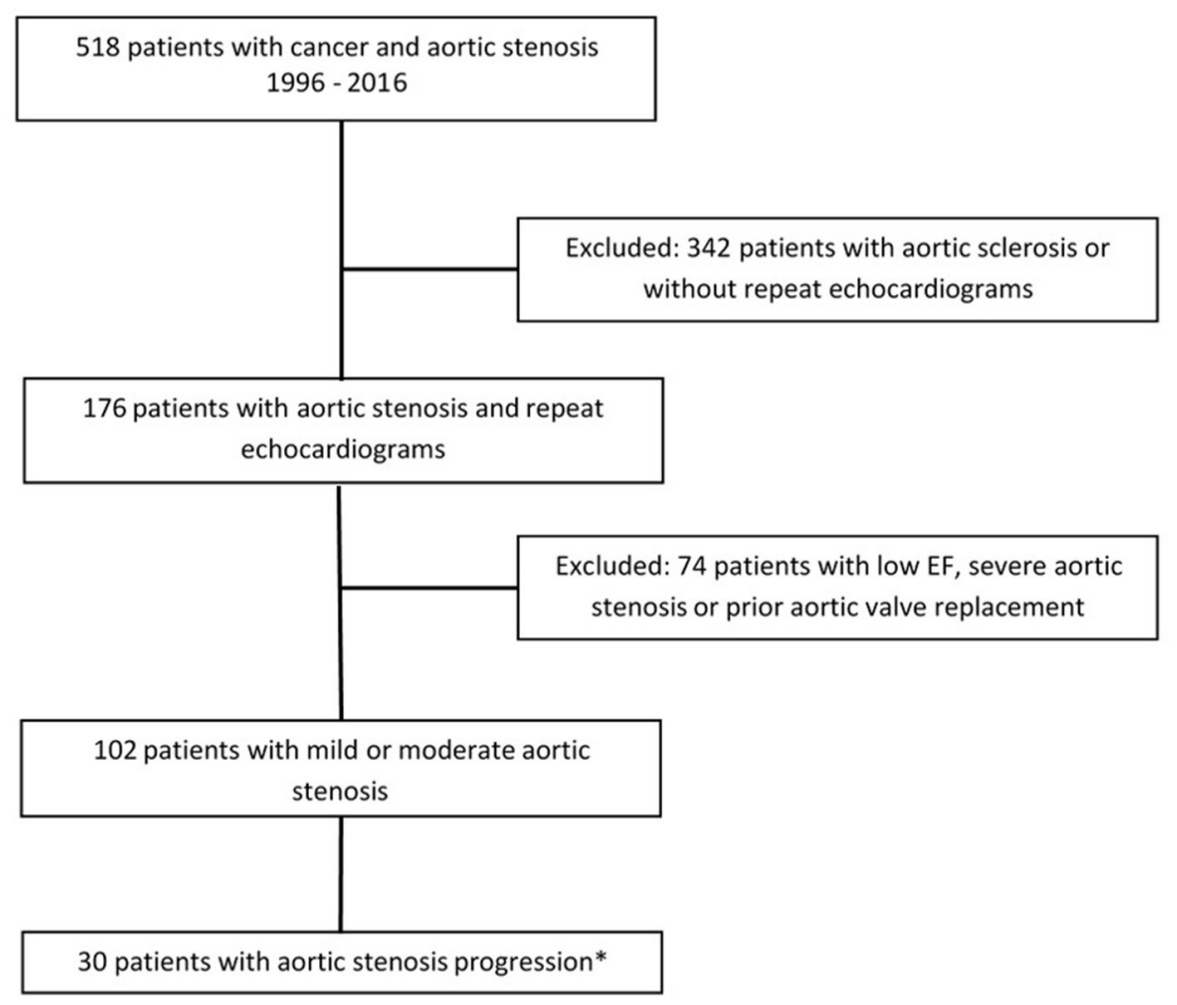
Study design. *Aortic stenosis progression defined as differential Vmax > 2 or mean gradient >20 mmHg or need for aortic valve replacement.

**Figure 2:**
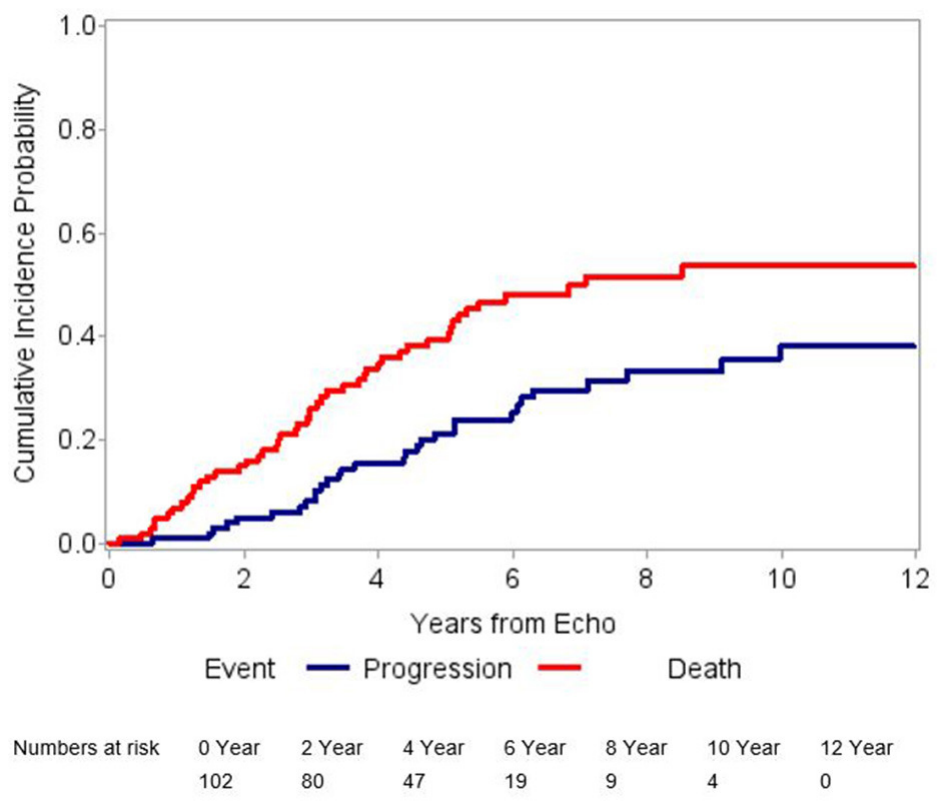
Graphical representation of cumulative incidence probabilities. The blue line shows the cumulative incidence of aortic stenosis progression after adjusting for competing risk. The red line shows the cumulative incidence of the competing risk event (death without aortic stenosis progression). This figure suggests a high incidence of a competing risk event.

Out of 518 patients identified with AS in our center between January 1st 1996 and December 31st 2016, 102 patients met the inclusion criteria (Figure 1). Twelve (12%) patients had mild AS and 90 (88%) patients, moderate AS. In addition to the baseline TTE, all patients included in our study had at least 1 follow-up TTE, whereas 57 (56%) had at least 2 and 30 (29%) had at least 3 TTEs performed at our institution. After a median follow-up time of 7.3 years (95% CI 6.3–9.4 years), 30 (29%) patients developed AS progression and 48 (47%) died without progression (Figure 2). Patient characteristics are summarized by progression status in Table 1. The median duration of follow-up for those with AS progression [4.0 years (IQR 2.5–6.3)] was longer to those who remained alive without AS progression [2.3 years (IQR 1.3–3.9)] or died without AS progression [1.9 years (IQR 1.1–3.3)]. The average annual rate of mean gradient change was 4.9 (SD 3.9) mmHg and annual rate of peak velocity change was 0.23 (SD 0.29) m/s for patients who developed AS progression. Among those with AS progression, 21 (70%) had AVR with 13 (62%) receiving surgical AVR and 8 (38%) transcatheter AVR (TAVR). Out of those receiving AVR, 10 (48%) died, [8 (80%) in the surgical AVR and 2 (20%) in the TAVR group] and their median survival after AVR was 4.2 years (95% CI, 2.1–10.2). No perioperative or periprocedural death occurred. Among those who had AS progression and did not receive AVR, 7 (78%) died and their median survival after AS progression was 1.4 years (95% CI, 0.2–3.4), which was significantly lower than the group receiving AVR (*p* = 0.0023).

**Table 1:** Baseline characteristics by progression status.

	**Progression status**			***P*-value**
	**Alive without AS progression (*N* = 24)**	**AS progression (*N* = 30)**	**Death without AS progression (*N* = 48)**	
Age (years)*	66.6 ± 7.7	66.5 ± 8.8	71.5 ± 8.8	**0.011**
Men (*n*, %)	9 (37.5)	17 (56.7)	27 (56.3)	0.268
CAD (*n*, %)	4 (16.7)	13 (46.4)	13 (27.1)	0.054
Hypertension (*n*, %)	20 (83.3)	23 (79.3)	33 (68.8)	0.335
Hyperlipidemia (*n*, %)	18 (75)	17 (58.6)	25 (52.1)	0.174
Diabetes mellitus (*n*, %)	10 (41.7)	12 (42.9)	12 (25)	0.188
Heart failure (*n*, %)	1 (4.2)	5 (17.9)	9 (18.8)	0.222
CVA (*n*, %)	7 (29.2)	1 (3.6)	2 (4.2)	**0.003**
COPD (*n*, %)	3 (12.5)	4 (14.3)	5 (10.4)	0.923
Chronic kidney disease (*n*, %)	5 (20.8)	12 (40)	19 (41.3)	0.205
Current smoker (*n*, %)	2 (8.3)	3 (10.3)	2 (4.3)	0.599
Solid tumor (*n*, %)	15 (62.5)	14 (48.3)	29 (60.4)	0.455
Advanced cancer (*n*, %)	13 (54.2)	20 (69)	28 (58.3)	0.505
Chest radiation (*n*, %)	6 (25)	10 (33.3)	13 (27.1)	0.765
Anthracyclines (*n*, %)	9 (37.5)	15 (50)	15 (31.9)	0.280
Cyclophosphamide (*n*, %)	9 (37.5)	17 (56.7)	11 (23.4)	**0.012**
Taxanes (*n*, %)	8 (33.3)	3 (10)	13 (27.1)	0.096
Tyrosine kinase inhibitors (*n*, %)	2 (8.3)	2 (6.7)	9 (18.8)	0.251
VEGF inhibitors (*n*, %)	1 (4.2)	0 (0)	2 (4.2)	0.602
HER2 antagonists (*n*, %)	3 (12.5)	0 (0)	1 (2.1)	**0.046**
Number of echocardiograms (*n*)*	3.1 ± 1.4	3.8 ± 1.7	2.9 ± 1.1	**0.043**
Interval between echocardiograms (months)*	35 ± 29	52 ± 32	26 ± 18	**<0.001**
Baseline ejection fraction (%)*	61.8 ± 5.1	60.4 ± 5.5	61.3 ± 5.9	0.605
Baseline aortic valve area (cm^2^)*	1.5 ± 0.4	1.4 ± 0.5	1.4 ± 0.4	0.617
Baseline mean gradient (mmHg)*	15.6 ± 5.4	17.8 ± 7.9	16.7 ± 7.5	0.456
Baseline maximal velocity (m/s)**	2.6 (2.4–2.9)	2.9 (2.3–3.4)	2.8 (2.3–3.1)	0.698
Chest pain (*n*, %)	2 (8.3)	8 (26.7)	6 (12.5)	0.155
Dyspnea (*n*, %)	3 (12.5)	13 (43.3)	12 (25)	**0.036**
Syncope (*n*, %)	2 (8.3)	3 (10)	5 (10.4)	1.000
Beta-blockers (*n*, %)	9 (37.5)	18 (64.3)	19 (39.6)	0.072
ACEI/ARB (*n*, %)	10 (41.7)	14 (48.3)	16 (33.3)	0.418
Diuretics (*n*, %)	7 (29.2)	10 (34.5)	19 (39.6)	0.677
Statins (*n*, %)	13 (54.2)	16 (53.3)	15 (31.3)	0.073
Anticoagulants (*n*, %)	3 (13)	8 (27.6)	6 (12.5)	0.266

*AS, Aortic stenosis; CAD, Coronary artery disease; CVA, cerebrovascular accident; COPD, Chronic obstructive pulmonary disease; VEGF, Vascular endothelial growth factor; HER2, Human epidermal growth factor receptor 2; ACEI/ARB, angiotensin converting enzyme inhibitor/angiotensin receptor blocker*.

* *Mean ± SD are presented*.

** *Median (interquartile range) are presented*.

*Bold values represent significant p values*.

Table 2 summarizes the results of univariate Fine-Gray analysis to identify associations with cumulative incidence of AS progression. Coronary artery disease (CAD), prevalent cyclophosphamide, dyspnea and beta-blocker use were associated with higher cumulative incidence of AS progression. CAD and beta-blocker use were correlated (*p* = 0.026). The use of VEGF inhibitors and HER2 inhibitors was associated with lower cumulative incidence of AS progression. After building a multivariable Fine-Gray model including age group, CAD and prevalent cyclophosphamide use; only CAD and prevalent cyclophosphamide use remained significant (Table 3A). Violation of the proportional hazards assumption was detected for prevalent cyclophosphamide use. Therefore, the subdistribution hazard ratio (sHR) was interpreted as the weighted average over follow-up in the model without time-dependent coefficient (14). The Fine-Gray model with a time-dependent coefficient for prevalent cyclophosphamide use (allowing two different sHRs for two time intervals, ≤3 years and more than 3 years of follow-up), is presented in Table 3B. The Fine-Gray model including a time-dependent coefficient for prevalent cyclophosphamide use with age group and CAD [sHR: 2.45 (95% CI, 1.19–5.04)], showed no significant association of prevalent cyclophosphamide for the time interval ≤3 years of follow-up [sHR: 1.17 (95% CI, 0.24–5.76)] and a significant association of prevalent cyclophosphamide use for the time interval >3 years follow-up [sHR: 3.81 (95% CI, 1.54–9.44)] on the cumulative incidence of AS progression (Table 3B).

**Table 2:** Univariate Fine-Gray model, aortic stenosis progression as an event of interest.

**Covariate**	**Level**	**sHR**	**95% CI**	***p*-value**
Age	In 1 Unit Change	0.97	(0.93–1.00)	0.059
Age group	>70 years	0.574	(0.281–1.173)	0.128
Sex	Women	0.78	(0.39–1.58)	0.487
Coronary artery disease	Yes	2.31	(1.12–4.77)	**0.024**
Hypertension	Yes	1.36	(0.56–3.26)	0.498
Diabetes mellitus	Yes	2.07	(0.99–4.34)	0.053
Current smoker	Yes	1.76	(0.66–4.74)	0.262
Heart failure	Yes	1.16	(0.48–2.83)	0.736
Cerebrovascular disease	Yes	0.53	(0.07–3.99)	0.541
Hyperlipidemia	Yes	0.96	(0.46–2.02)	0.923
Chronic kidney disease	Yes	1.17	(0.56–2.44)	0.673
Advanced cancer	Yes	1.57	(0.72–3.40)	0.255
Prevalent chest radiation	Yes	1.76	(0.76–4.04)	0.185
Incident chest radiation	Yes	0.51	(0.19–1.32)	0.163
Prevalent anthracyclines	Yes	1.64	(0.78–3.43)	0.189
Incident anthracyclines	Yes	1.52	(0.70–3.27)	0.289
Prevalent cyclophosphamide	Yes	3.01	(1.53–5.94)	**0.001**
Incident cyclophosphamide	Yes	1.47	(0.64–3.35)	0.364
Taxanes	Yes	0.33	(0.10–1.11)	0.072
Tyrosine kinase inhibitors	Yes	0.46	(0.11–1.93)	0.287
VEGF inhibitors	Yes	<0.001	(0.00–0.00)	**<0.001**
HER2 antagonists	Yes	<0.001	(0.00–0.00)	**<0.001**
Other chemotherapy	Yes	0.75	(0.36–1.56)	0.441
Calcium	In 1 Unit Change	1.03	(0.61–1.73)	0.909
Phosphorus	In 1 Unit Change	0.82	(0.51–1.32)	0.415
Creatinine	In 1 Unit Change	0.85	(0.60–1.22)	0.379
Chest pain	Yes	2.22	(0.99–4.97)	0.053
Dyspnea	Yes	2.07	(1.02–4.20)	**0.043**
Syncope	Yes	0.99	(0.31–3.16)	0.986
Beta-blockers	Yes	2.52	(1.19–5.33)	**0.016**
ACEI/ARB	Yes	1.79	(0.88–3.65)	0.111
Diuretics	Yes	1.04	(0.48–2.23)	0.926
Statins	Yes	1.64	(0.81–3.33)	0.173
Anticoagulants	Yes	1.96	(0.91–4.235)	0.087

*sHR, subdistribution hazard ratio; CI, confidence interval; VEGF, Vascular endothelial growth factor; HER2, Human epidermal growth factor receptor 2; ACEI/ARB, angiotensin converting enzyme inhibitor/angiotensin receptor blocker. Bold values represent significant p values*.

**Table 3:** Multivariable Fine-Gray model, aortic stenosis progression as an event of interest.

**Covariate**	**sHR**	**95% CI**	***p*-value**
**A**. Without time-dependent coefficient for prevalent cyclophosphamide*			
Age > 70 years	0.71	(0.31–1.61)	0.409
Coronary artery disease	2.46	(1.21–5.00)	**0.013**
Prevalent cyclophosphamide	2.76	(1.25–6.09)	**0.012**
**B**. With time-dependent coefficient for prevalent cyclophosphamide**			
Age > 70 years	0.71	(0.32–1.60)	0.409
Coronary artery disease	2.45	(1.19–5.04)	**0.023**
Prevalent cyclophosphamide use for the time interval ≤3 years of follow-up	1.17	(0.24–5.76)	0.841
Prevalent cyclophosphamide use for the time interval >3 years of follow-up	3.81	(1.54–9.44)	**0.004**

*sHR, subdistribution hazard ratio; CI, confidence interval*.

* *When, age group, coronary artery disease, and prevalent cyclophosphamide are in the multivariable model, coronary artery disease and prevalent cyclophosphamide remained significant*.

** *When, age group, coronary artery disease, and prevalent cyclophosphamide are in the multivariable model, coronary artery disease and prevalent cyclophosphamide for the time interval >3 years follow-up remained significant. Bold values represent significant p values*.

The univariate Fine-Gray analysis to identify associations with cumulative incidence of death revealed that age >70 years and use of a tyrosine kinase inhibitor were associated with higher cumulative incidence of death. Use of prevalent cyclophosphamide and statins were associated with lower cumulative incidence of death. After building a multivariable Fine-Gray model including age group, prevalent cyclophosphamide, and statin use; prevalent cyclophosphamide use remained significant (Table 4). Proportional hazards assumption was satisfied for all variables included in the multivariable model for death.

**Table 4:** Multivariable Fine-Gray model, death without aortic stenosis progression as an event of interest.

**Covariate**	**sHR**	**95% CI**	***p*-value**
Age > 70 years	1.87	0.99–3.56	0.055
Prevalent cyclophosphamide	0.27	0.08–0.87	**0.029**
Statins	0.57	0.31–1.05	0.069

*sHR, subdistribution hazard ratio; CI, Confidence interval*.

*When age group, prevalent cyclophosphamide, and statins are in the multivariable model, prevalent cyclophosphamide remained significant. Age and statins were marginally significant. Bold values represent significant p values*.

The first sensitivity analysis (Supplementary Table 1) using rate of progression by mean gradient demonstrated no significant differences among subgroups. However, those with hematologic malignancies had a non-significant higher rate of progression than those with solid tumors (3.75 ± 4.51 vs. 2.06 ± 4.14; *p* = 0.052). The second sensitivity analysis using rate of progression by jet velocity (Supplementary Table 2) demonstrated no significant differences among subgroups.

## Discussion

In our study of patients with cancer, mild or moderate AS, and normal LV systolic function who underwent serial clinical and echocardiographic evaluation at our tertiary referral center, we demonstrated that in those who develop AS progression the average annual rate of mean gradient change was 4.9 (SD 3.9) mmHg and annual rate of peak velocity change was 0.23 (SD 0.29) m/s. AS progression was associated with CAD as well as cyclophosphamide use in a time-dependent fashion. Notably, patients more frequently died without AS progression and death occurred as early as in the first year of follow-up, suggesting that patients with cancer and AS experience increased risk of death from non-valvular causes (15).

**Figure 3:**
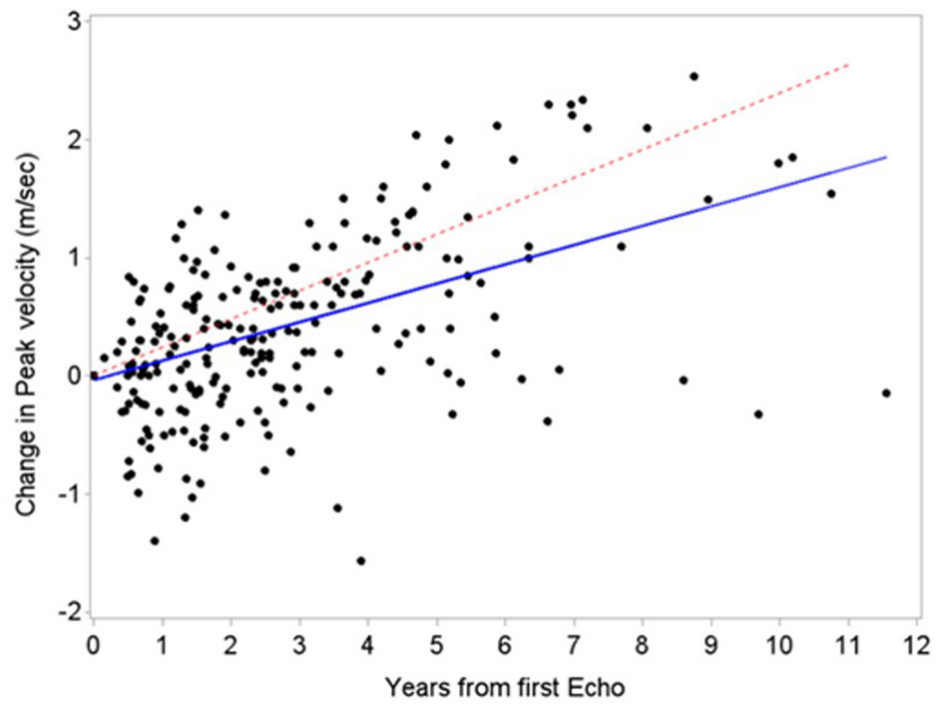
Comparison of aortic stenosis progression between patients with cancer and a historical cohort. Similar aortic stenosis progression based on peak velocity between patients with cancer (blue line, estimated slope 0.16 m/s/year) and a historical cohort by Rosenhek et al. (18) (red line, estimated slope 0.24 m/s/year).

The annual rate of mean gradient and peak velocity change in our cohort (4.9 -SD 3.9- mmHg and 0.23 -SD 0.29- m/s, respectively) was similar to the general population based on historical (16–18) and contemporary (15) cohorts reporting an annual increase in mean gradient and peak velocity of 7 to 8 mmHg and 0.32 -SD 0.34- to 0.45 -SD 0.38-m/s, respectively (Figure 3). This was also noted among our high-risk subgroups such as those receiving chest radiation, who had mean gradient changes of 3.11 -SD 3.21- mmHg and peak velocity changes of 0.18 -SD 0.26- m/s. Current surveillance guidelines recommending repeat echocardiograms in patients with mild or moderate AS every 3 to 5 and 1 to 2 years, respectively (6). This may still be applicable to the cancer population, however in those with high competing risk of death we suggest personalizing timing of interval progression surveillance based on a risk-benefit discussion.

Univariate and multivariable analyses showed a potentially novel association between prevalent cyclophosphamide exposure and the cumulative incidence of AS progression. In the pathogenesis of AS, upregulation of transcription factors such as nuclear factor-Kappa B and hypoxia-inducible factor-2 leads to migration of endothelial progenitor cells from the ventricular and aortic surfaces into the middle leaflet layer, which subsequently results in proliferation, neoangiogenesis (19, 20), and intra-leaflet hemorrhage (21). We hypothesize that cyclophosphamide exposure can potentiate intra-leaflet hemorrhage in affected valves, and this can promote further inflammation, worsening valve stenosis. This process may be slow over time as noted in our multivariable analysis. Further studies are needed to confirm this association and postulated mechanism.

We found an association between lower cumulative incidence of AS progression with use of VEGF inhibitors or HER2 inhibitors. Given that the total number of patients receiving VEGF inhibitors and HER2 inhibitors was very small in our cohort (3 and 4 patients, respectively), we decided not to include these variables into our multivariable model to avoid further inferences. Despite this, the potential protective effect of VEGF inhibitors and HER2 inhibitors against AS progression may have biological plausibility given that they could decrease neoangiogenesis and collagen I overexpression (the most common type of collagen in the aortic valve) (22–25) which play a role in the pathogenesis of AS. Future studies need to elucidate this potential association.

Similar to the general population (26), an association between CAD and the cumulative incidence of AS progression was found in our study. We did not identify specific individual risk factors associated with AS progression, however our small sample size, similar distribution of risk factors and inability to account for disease control may affect our results. Additionally, as previously demonstrated in the ASTRONOMER (27) and SEAS (28) randomized clinical trials, we observed that statin use did not ameliorate cumulative incidence of AS progression. However, significant statin protection against death was found in our univariate analysis but this was not confirmed in our multivariable analysis. The potential role of statins in mortality prevention among patients with Łcancer and concomitant CAD needs to be further explored (29).

Currently, TAVR has produced a paradigm shift in the treatment of severe AS due to its proven lower periprocedural risk when compared with surgical AVR. Our cohort of patients spanned a 20 year period most of which was prior to the TAVR era thus leading to the majority of patients receiving surgical AVR. One retrospective study in patients with cancer and severe AS suggested better survival after TAVR when compared to those not receiving AVR or receiving surgical AVR, however, this is considerably lower than the general population, and in about half of the cases did not exceed 1 year (30). A complex decision-making process involving performance status, comorbid conditions, and expected improvement in the quality of life is required to identify those patients with AS progression who will benefit from this procedure.

Our study has several limitations. First, due to its retrospective nature, causal associations cannot be made, cause of death could not be accurately determined, and selection bias could be present (i.e., those who were thought to have better prognosis underwent echocardiographic surveillance more often). We conducted this study at a large tertiary care cancer center, which carries the risk of referral bias. The significance of outcomes may be affected by median follow-up, a higher number of repeat echocardiograms in the group with AS progression, and cohort size.

In conclusion, patients with mild to moderate AS and cancer are more likely to die before having progression of valvular stenosis. CAD and prevalent cyclophosphamide use for the time interval >3 years of follow-up are significantly associated with AS progression.

## Data Availability Statement

The raw data supporting the conclusions of this article will be made available by the authors, without undue reservation.

## Ethics Statement

The studies involving human participants were reviewed and approved by MD Anderson Cancer Center IRB. Written informed consent for participation was not required for this study in accordance with the national legislation and the institutional requirements.

## Author Contributions

KB-J: idea conception, study design, data collection, analysis, manuscript preparation, and revision of final manuscript. NP: study design, data collection, analysis, manuscript preparation, and revision of final manuscript. JB: study design and revision of final manuscript. NA, AA, and SG: data collection, manuscript preparation, and revision of final manuscript. JS: analysis, manuscript preparation, and revision of final manuscript. SH, CI, and AD: analysis and revision of final manuscript. SY: study design, data collection, analysis, and revision of final manuscript. All authors contributed to the article and approved the submitted version.

## Conflict of Interest

The authors declare that the research was conducted in the absence of any commercial or financial relationships that could be construed as a potential conflict of interest.
