# Supplementary material for: Rate of Progression of Aortic Stenosis in Patients With Cancer
Source: Front Cardiovasc Med. 2021 Mar 18;8:644264. doi: 10.3389/fcvm.2021.644264 (PMC8012898; doi:10.3389/fcvm.2021.644264)
Supplement: Supplementary file 1 [file Data_Sheet_1.pdf]

Supplementary Table A.

Changes in mean gradient according to high-risk groups

Subgroup	Group 1	Group 2	P-value
Age (<=70 vs >70)	2.86 ± 3.90	2.75 ± 4.81	0.894
Gender (male vs female)	3.20 ± 4.57	2.38 ± 4.15	0.345
Cancer (hematologic vs solid)	3.75 ± 4.51	2.06 ± 4.14	0.052
Stage (low vs high)	2.92 ± 4.05	2.73 ± 4.63	0.827
Chest radiation (no vs yes)	2.69 ± 4.77	3.11 ± 3.21	0.607

Supplementary Table B.

Changes in peak velocity according to high-risk groups

Subgroup	Group 1	Group 2	P-value
Age (<=70 vs >70)	0.16 ± 0.39	0.18 ± 0.34	0.836
Gender (male vs female)	0.19 ± 0.36	0.15 ± 0.38	0.570
Cancer (hematologic vs solid)	0.21 ± 0.45	0.14 ± 0.28	0.329
Stage (low vs high)	0.14 ± 0.41	0.19 ± 0.34	0.557
Chest radiation (no vs yes)	0.16 ± 0.40	0.18 ± 0.26	0.757
